# LIPSS Structures Induced on Graphene-Polystyrene Composite

**DOI:** 10.3390/ma12213460

**Published:** 2019-10-23

**Authors:** Dominik Fajstavr, Klára Neznalová, Václav Švorčík, Petr Slepička

**Affiliations:** Department of Solid State Engineering, University of Chemistry and Technology Prague, 166 28 Prague, Czech Republic; D.Fajstavr@seznam.cz (D.F.); klara.neznalova@vscht.cz (K.N.); vaclav.svorcik@vscht.cz (V.Š.)

**Keywords:** nanocomposites, polymers, laser exposure

## Abstract

A laser induced periodic surface structure (LIPSS) on graphene doped polystyrene was prepared by the means of a krypton fluoride (KrF) laser with the wavelength of 248 nm and precisely desired physico-chemical properties were obtained for the structure. Surface morphology after laser modification of polystyrene (PS) doped with graphene nanoplatelets (GNP) was studied. Laser fluence values of modifying laser light varied between 0–40 mJ·cm^−2^ and were used on polymeric PS substrates doped with 10, 20, 30, and 40 wt. % of GNP. GNP were incorporated into PS substrate with the solvent casting method and further laser modification was achieved with the same amount of laser pulses of 6000. Formed nanostructures with a periodic pattern were examined by atomic force microscopy (AFM). The morphology was also studied with scanning electron microscopy SEM. Laser irradiation resulted in changes of chemical composition on the PS surface, such as growth of oxygen concentration. This was confirmed with energy-dispersive X-ray spectroscopy (EDS).

## 1. Introduction

Polystyrene (PS) is one of the most widely used plastic materials, having applications in industries of electronics, automobiles, packaging, appliances, construction, etc. [1]. However, PS is only rarely used in a “pure” form, because many additives are added to it to utilize its chemical or mechanical properties. One way to provide this polymeric material with new modified properties is to create a composite with different nanoparticles. At present, we often encounter efforts to create Conductive Polymeric Nanocomposites. These merge the conductive properties of the nanoparticles with the non-conductive properties of the polymeric substrate [2]. The advantage of these composites is a possibility to change properties of studied material such as surface morphology, electrical conductivity, or roughness [3,4]. For the manufacturing of sensors, it is desirable that such a composite changes its electrical properties in response to changes in external influences such as chemical, thermal, or mechanical inputs [5]. Surface coatings of the biopolymer can be modified by various methods using grafting [6], laser modification techniques [7,8,9], or plasma [10].

Laser exposure of polymer substrates can induce the formation of so-called laser-induced periodic surface structures (LIPSS) [11,12,13]. Periodic patterns may be divided into two subgroups based on the relation of their period to the laser wavelength. The first group that has a period comparable with the wavelength of the incident laser beam is described as low spatial frequency LIPSS, while the group whose period is much smaller than the wavelength of the laser beam is described as high spatial frequency LIPSS. The former structures from the first group are most commonly observed on polymer substrates, while the latter are more often formed on laser-treated semiconductors and metals [14]. Low-intensity LIPSS on polymer foils were described as oriented along the main axis of the polarization of the laser beam [15]. A good absorption by the substrate in the wavelength region of the laser radiation is necessary. Therefore, to fulfil these conditions we have observed these structures on polymers with strongly absorbing groups, such as aromatic rings and systems of conjugated bonds. The particular mechanism of the formation of the ripples is a complex phenomenon, though a broadly accepted theory is that the major influence to the ripple-forming process has an interference between a primary (incident) beam and a secondary (perpendicularly reflected) beam. This effect causes local accumulation of energy, resulting in the non-crystalline phase of the polymer substrate being temporarily heated above the glass transition temperature and the crystalline one being melted [16,17]. These effects are responsible for the material transfer, the flow from the high-temperature areas to the lower-temperature ones, thus creating a periodic pattern on the polymer surface [18]. The propagation of interfered surface wave induces the inhomogeneous heat intensity and thus mass transfer of polymer, which is strongly affected by the presence of graphene nanoplatelets (GNP) in the polymer bulk. The influence of graphene nanoplatelets is mostly based on the fact that the heat conductivity is higher for the GNP in comparison with the polymer chain.

The specific surface morphology and pattern type has an extremely important role in cell-substrate interaction [19], where the chemical and morphological properties of these structures were confirmed to influence in vitro tailoring of protein adsorption and cell behavior. The ultrashort laser pulses were applied to biomaterial composites for construction of biomimetic surface [20]. LIPSS have been applied for enhancement of mesenchymal cell growth and adhesion. The smart design of polymer influenced the cell alignment which was cell-type dependent, human myoblasts was proved to influence the alignment for certain periodicities [17]. The migrations of the cells was successfully guided with the character of the cells to migrate towards the microstructures [21]. Created periodic pattern which can be subsequently metallized was also used for construction of sensors [22], antibacterial surfaces [23], or for cell guidance [24].

In recent studies, we can see composites of graphene materials as a promising element in the field of nanosciences [25]. The basic characteristic of graphene is that it is a single layer of carbon atoms that are simply arranged. Another essential feature is that like others zero-gap semiconductors it has excellent electrical conductivity. This is caused by a field-generated band gap and high electron mobility [26]. Processes of preparation of a graphite composite can be both chemical and laser-driven for the preparation of desired nanomaterial. However, graphene is difficult to synthesize at high pressures and temperatures inside the plasma, making the above methods more practical for the formation of quantum dots and nanoparticles [27,28]. At high temperatures, carbon structures also become amorphous, causing complete disappearance of the hexagonal lattice [29].

The aim of this work was to focus on how to incorporate graphene nanoplatelets (GNP) into polystyrene (PS) layers and prepare them for subsequent laser modification. Various analytical methods were designed to study the effect of the presence of graphene nanoparticles on the physico-chemical properties of prepared substrates and on surface properties such as: surface roughness, morphology, chemistry, and surface area. To the best of our knowledge, this is the first time that LIPSS structures doper with graphene was prepared using a combination of doping and excimer exposure.

## 2. Materials and Methods

### 2.1. Materials, Procedures and Apparatus

The surface structures have been modified with use of KrF excimer pulse laser (Coherent, Inc., Santa Clara, USA, COMPexPro 50F, 248 nm wavelength, 20–40 nanoseconds pulse duration and 10 Hz repetition rate) on polystyrene (PS) foil with biaxial orientation (thickness of 50 µm, supplied by Goodfellow Ltd.). The laser beam has been polarized linearly by a ultraviolet-grade silica fused cube 25 × 25 × 25 mm^3^ with layer of active polarization (model PBSO-248-100). In order to achieve even irradiation of the substrates, an iris was inserted into the optical system through which only the central part of the laser beam (0.5 × 1.0 cm^2^) with an already homogeneous energy distribution passed [29]. It is known from previous studies that the best uniformity of surface structures was prepared with 6000 laser pulses [12], so in this study we treated all samples with this number of pulses and laser fluences in interval 0–40 mJ·cm^−2^.

For synthesis of polymeric layers with integrated graphene nanoplatelets (GNP) we used solvent casting method. The pristine polymeric polystyrene (PS) was put into toluene to dissolve in a suitable amount to achieve required layer thickness. The solvent is evaporated in a fume cupboard under conditions of atmospheric pressure, with a temperature about 22 °C for 9 h. The GNP materials have been supplied from Goodfellow, UK. For homogeneous distribution of the dispersion in solution, the GNP was already modified by the supplier with an argon plasma discharge. The desired quantity of GNP has been weighed for the preparation of appropriate solutions in a range from 0 to 40 wt. %.

### 2.2. Characterization Techniques

The morphology of the surface has been studied with Dimension ICON atomic force microscope (Bruker Corp., Billerica, MA, USA) and to determine the samples, we used QNM mode for analysis. Silicon apex mounted on Nitride Lever SCANASYST-AIR and the constant of the spring was 0.4 N/m was used. All atomic force microscopy (AFM) results were acquired with software NanoScope Analysis (version 1.80, Bruker Corp., Billerica, MA, USA). Measured and calculated mean roughness values (R_a_) indicate how much they differ on average from the mean surface plane.

Scanning electron microscopy (SEM) was used for surface imaging by FIB-SEM microscope (Tescan, LYRA3 GMU, Brno, Czech Republic). It has been used with a voltage of 5 kV for acceleration. All studied samples had to be sputtered with metal in order to make the surface electrically continuous. We used platinum (99.9995% purity, SAFINA, Vestec, Czech Republic) and the thickness of the Pt layer was 20 nm. For metal coverage diode, a sputtering technique was chosen (Q300T Quorum, Quorum Technologies Ltd., Laughton, United Kingdom). The chemical composition and atomic representation were investigated by energy dispersive X-ray spectroscopy (EDS, X-MaxN analyzer, SDD detector 20 mm^2^, Oxford Instruments, Abingdon-on-Thames, UK). Voltage acceleration of SEM-EDS technique was set up to 5 kV.

Contact angle was determined by goniometry with the static water drop method. The measurements were performed using distilled water (six different positions) and the Surface Energy Evaluation System (SEE System, Advex Instruments, Brno, Czech Republic) was used. By Automatic pipette the water drop of volume (2.0 ± 0.1) mL was deposited on the polymer’s surface and the captured photo was evaluated. The measurement was carried out at room temperature. Samples were measured immediately after modification.

The electrical discontinuity/continuity of studied samples was examined by measuring electrical sheet resistance (Rs). For determination of Rs by standard Ohm’s method using KEITHLEY, a 487 pico-ampermeter was used (Tektronix U.K Limited, Oldbury, United Kingdom). Two Au contacts (50 nm thick) were sputtered on the layer surface for resistance measurement. Typical error of the measurement was ±5%.

## 3. Results

### 3.1. Surface Roughness and Morphology

**Figure 1:**
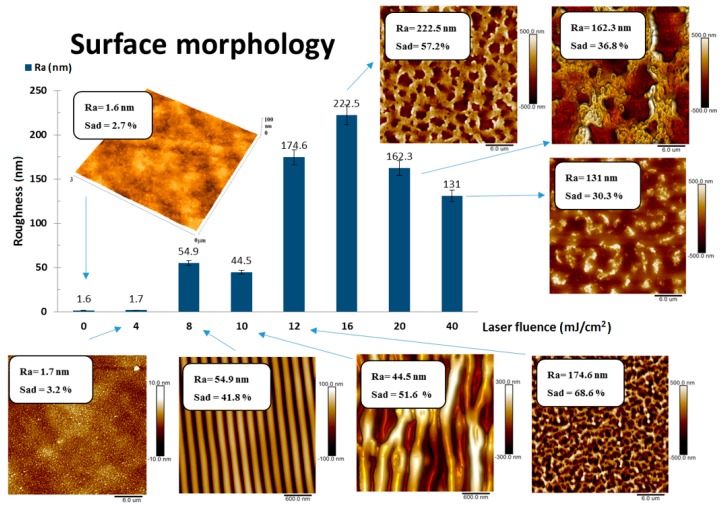
The dependence of surface roughness on modifying laser fluence values (6000 pulses) of polystyrene (PS) samples doped with 40 wt. % of graphene nanoplatelets (GNP). Atomic force microscopy (AFM) images (3 × 3 μm^2^ and 30 × 30 μm^2^) include values of Sad (surface area difference) and Ra (roughness of the surface).

Figure 1 shows the surface roughness values and its corresponding AFM images for better understanding of structural growth on the surface. The illustrated samples contain 40 wt. % GNP, and a surface morphology of samples with 10, 20, and 30 wt. % exhibited a very similar surface morphology, therefore only this set of samples was chosen for demonstration. All samples were modified with 6000 laser pulses.

The optimal periodic pattern was attained by laser fluence of 8 mJ cm^−2^, where we observed periodical ripple formation that is typical for aromatic polymers modified with an excimer laser beam. Its surface exhibited a roughness of 54.9 nm. Exposure with lower laser fluence did not induce the LIPSS, instead a globular nanostructure appeared. However, a higher energy of laser beam induced different periodical patterns on doped polystyrene surface. It was observed that 10 mJ cm^−2^ of laser fluence value is still sufficient to induce a pattern where the ripple structures (LIPSS) dominate, but as the same time they are not as parallel as in the case of 8 mJ cm^-2^ and also we can observe how individual ripples connect with each other. Laser fluence value of 12 mJ cm^−2^ creates a surface with dominant type of structure of web or net instead of ripples. In the case of 16 mJ cm^−2^ “threads” in the so called-net structure are thicker and in the case of 20 mJ cm^−2^, even more so. Creation of this net structure was accompanied by a sudden rise of surface roughness values, where the maximum 222.5 nm was reached for the fluence of 16 mJ cm^−2^. To achieve the highest value of laser fluence 40 mJ cm^−2^ polarizer had to be removed and therefore the sample modified with this non-polarized laser beam exhibited collapsed structures with no periodicity or orientation. A similar experiment was performed by Rebollar et al. [30], where LIPSS structures have been successfully prepared on expanded graphite introduced in the poly (ethylene terephtalate) matrix.

**Figure 2:**
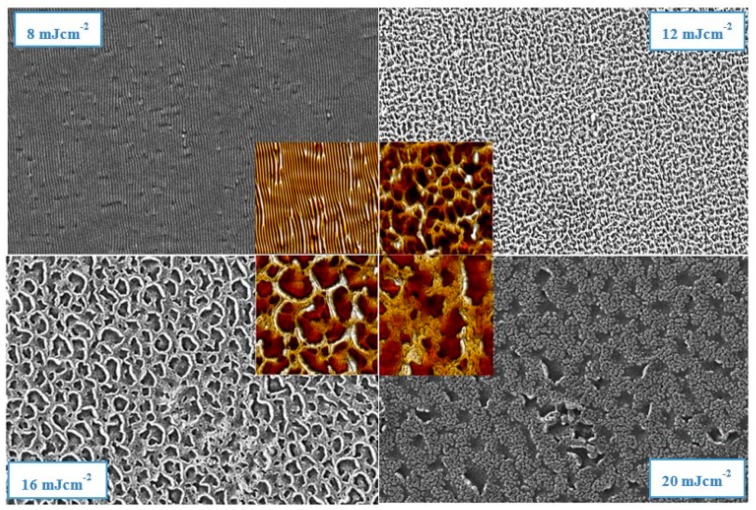
The scanning electron microscopy (SEM) of surface morphology of PS polymeric substrate doped with 40 wt. % of GNP after laser modification with corresponding laser fluence values. The SEM area is 50 × 50 microns, the AFM area in the center is 10 × 10 μm^2^.

Figure 2 shows scans of SEM results and for comparison AFM scans are included as well. This analytical method was used to examine the homogeneity of structures because the view field of the scanned area was chosen to be larger than the AFM images. SEM scans confirmed the creation of a ripple structure after an 8 mJ cm^−2^ modification. However, a larger area revealed spots of deflection from the ideal parallel orientation of the ripples. Modifying with 12 mJ cm^−2^ seemed to prepare the most homogenous surface without any defects in a created “web” structure. Treatment with laser fluence at 16 mJ cm^−2^ and 20 mJ cm^−2^ provided confirmation of the same structure measured with AFM as well.

**Figure 3:**
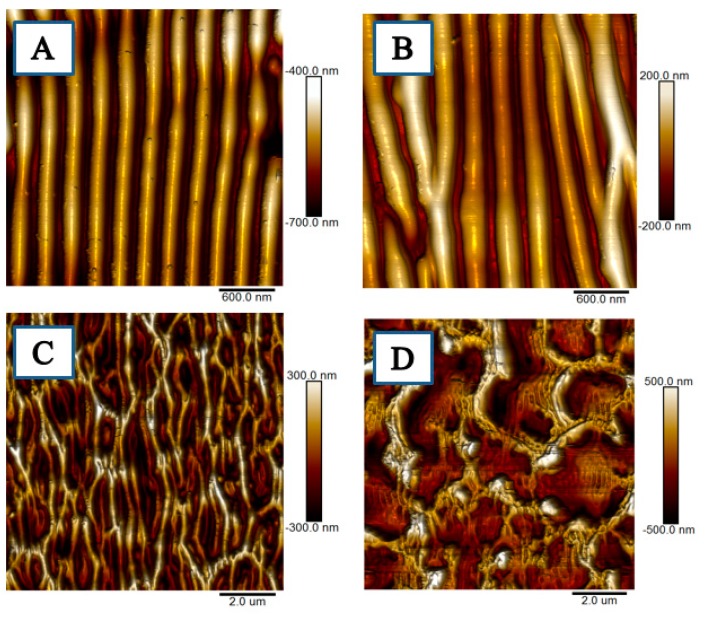
AFM scans (3 × 3 μm^2^ or 10 × 10 μm^2^) of pristine polystyrene modified with 6000 pulses and laser fluence values: (**A**) 8, (**B**) 10, (**C**) 12, and (**D**) 16 mJ·cm^−2^.

For comparison, we also included the surface morphology of laser treated polystyrene foils without graphene nanoplatelets (Figure 3). As was expected, pristine polystyrene foils prepared by the same method ad doped with graphene nanoplatelets exhibited a similar in their periodic pattern formation ability, also the laser fluence 12 mJ·cm^−2^ was acceptable for both type of foils as the threshold where the periodic pattern collapsed to a less uniform structure. The structure collapse was more pronounced for GNP doped foils, probably due to the different thermal conductivity of polymer layer enriched with GNPs, which affects the structure formation to a greater degree.

### 3.2. Electrical Properties and Wettability

**Figure 4:**
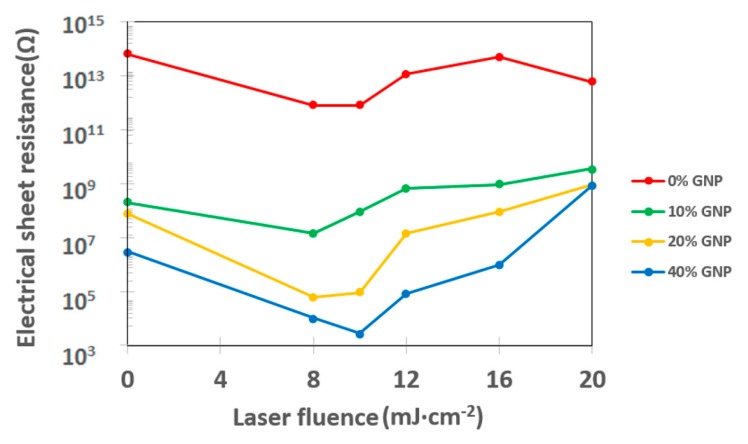
Dependence of sheet electrical resistance on laser fluence values after modification of polystyrene samples.

We also examined the sheet resistance of laser treated polystyrene substrates. As it is evident from Figure 4, as expected, with an increasing amount of GNPs, the surface resistance decreased. This decrease is more pronounced between pristine polystyrene substrate and substrate doped with 10 wt. % of graphene nanoplatelets. More surprisingly, the sheet resistance was strongly affected by the applied laser fluence onto both pristine and GNP doped polystyrene. For lower laser fluences, the sheet resistance decreased with increasing fluence. The minimal resistance values were achieved for fluences between 8 and 10 mJ·cm^−2^. In contrast, increasing of laser fluence above this interval led to an increase of the sheet resistance. Here we observed an interesting phenomenon. Firstly, even for highly doped samples, the sheet resistance was “relatively” high, which was caused by the composite character of the sample. Also, the increase of sheet resistance was caused by a similar reason, since during laser exposure with higher fluences the surface structure collapses, making distribution of GNPs less homogeneous, thus the sheet resistance increases.

**Figure 5:**
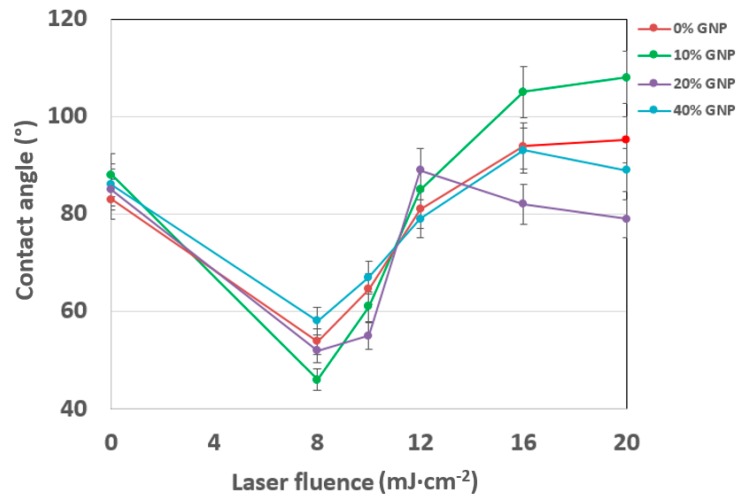
The dependence of contact angle on laser fluence values after modification of polystyrene samples doped with a different amount of GNP.

As it is obvious from Figure 5, the amount of graphene nanoplatelets did not influence the wettability of non-treated samples significantly. Applied laser fluence 8 mJ·cm^−2^ onto polystyrene samples induced a significant decrease of the contact angle, thus increasing the wettability of samples. Subsequent increase of laser fluences induced a steady increase of the contact angle, reaching the values above the non-treated samples, with an exception of a sample doped with 20 wt. % of GNPs, where the value remained almost similar to the pristine level at 20 mJ·cm^−2^.

### 3.3. Analysis of Surface Chemistry

**Figure 6:**
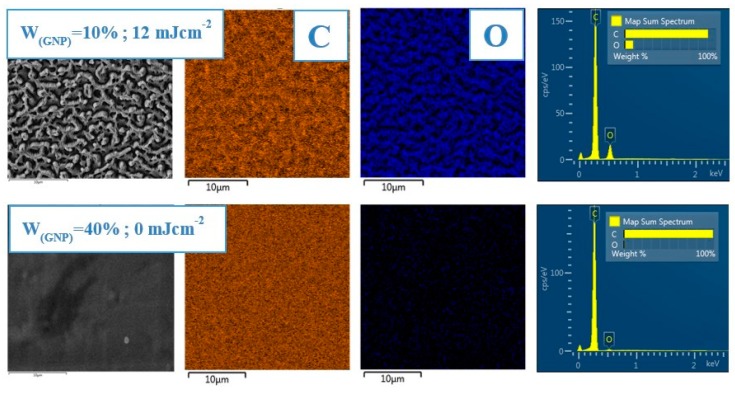
Selected EDS element distribution maps of C and O on PS polymer treated with a laser fluence value of 12 mJ·cm^−2^ (6000 pulses), doped with 10% GNP and non-modified with 40% GNP. View field of 30 × 30 μm^2^.

To determine the chemical composition of the prepared samples, the EDS method was used. Oxygen and carbon were investigated in their weight concentrations on the substrates, which underwent exposure with laser fluence values from 8 to 40 mJ·cm^−2^, and on the non-modified samples which were exposed to a different amount of GNPs varying from 10 to 40 wt. %. The element mapping (O, C) is also documented in Figure 6. There we can see homogenous distribution of elements on the non-modified surface and for comparison the scans of laser modified samples are also included, where we can observe a fit of element concentration corresponding to the created patterns. It can be seen from the data in Table 1 that all non-modified samples contain more than 99 wt. % of carbon. These samples differed in the C/O ratio composition between each other only very slightly and the amount of detected oxygen moved between 0.66% and 0.72% for samples prepared with 10, 20, 30, and 40 wt. % of GNP. Rather different results were observed after analysis of samples modified with an excimer laser. For further analysis we have chosen samples prepared with 10 wt. % and they were treated with laser fluence values of 8, 16, 20, and 40 mJ·cm^−2^. Excimer exposure led to a significant increase in detected oxygen. Modification with laser fluence 8 mJ·cm^−2^ induced a surface with the highest amount of oxygen (wt. % = 10.97 ± 0.08). Energy of 16 mJ·cm^−2^ led to the lowest concentration of oxygen (wt. % = 7.11 ± 0.07) and elemental concentration for modification with higher laser fluence (20 and 40 mJ·cm^−2^) did not change from this interval. The explanation of why laser modification leads to such an increase in oxygen concentration in the sample material is related with mechanisms that were mostly prepared immediately after laser treatment. A common feature is the increase in oxygen concentration over time when the samples are exposed to the surrounding atmosphere. This phenomenon is often documented after plasma or laser modification. Subsequently, chemical processes such as attack of the polymer chain by atmospheric oxygen or other radicals can result in the creation of a new functional oxygen groups (carboxylic acid, aldehyde, alcohol, and ketone) [6,10].

## 4. Conclusions

Modification with an excimer laser of polystyrene substrate doped with GNP leads to the creation of laser induced periodic surface structures (LIPSS). The optimal periodic pattern was achieved for modification with a laser fluence value of 8 mJ·cm^−2^ which induces the formation of parallel oriented ripples. Modification with higher fluence supports the formation of a web structure which subsequently collapses due to modification with 40 mJ·cm^−2^. Different concentrations of doped GNP did not seem to have a significant role in polystyrene surface morphology for samples without treatment. EDS analysis revealed that the laser modification induces significant differences in the atomic composition of studied materials and due to their interaction with surrounding atmosphere, as a dramatic growth of concentration of oxygen surface was measured on the surface. The sheet resistance of treated GNP is strongly affected by the applied laser fluence onto both pristine and GNP doped polystyrene. The wettability of both pristine and doped samples was also significantly affected by the laser fluence, while the amount of GNP without the treatment did not change either the resistance and wettability significantly.

## Figures and Tables

**Table 1:** Concentration of elements (wt. %) measured with EDS spectroscopy on polymer PS doped with 10%–40% GNP or samples doped with 10% of GNP treated with a laser fluence of 8–40 mJ·cm^−2^. View field of 30 × 30 μm^2^.

Laser Modified Samples (W_(GNP)_ = 10%)			Non-Modified Samples		
E (mJ cm^−2^)	C (wt. %)	O (wt. %)	GNP (wt. %)	C (wt. %)	O (wt. %)
8	89.03 ± 0.08	10.97 ± 0.08	10	99.29 ± 0.03	0.71 ± 0.03
16	92.89 ± 0.07	7.11 ± 0.07	20	99.30 ± 0.04	0.70 ± 0.04
20	90.98 ± 0.07	9.02 ± 0.07	30	99.28 ± 0.07	0.72 ± 0.07
40	91.32 ± 0.04	8.68 ± 0.04	40	99.34 ± 0.04	0.66 ± 0.04
